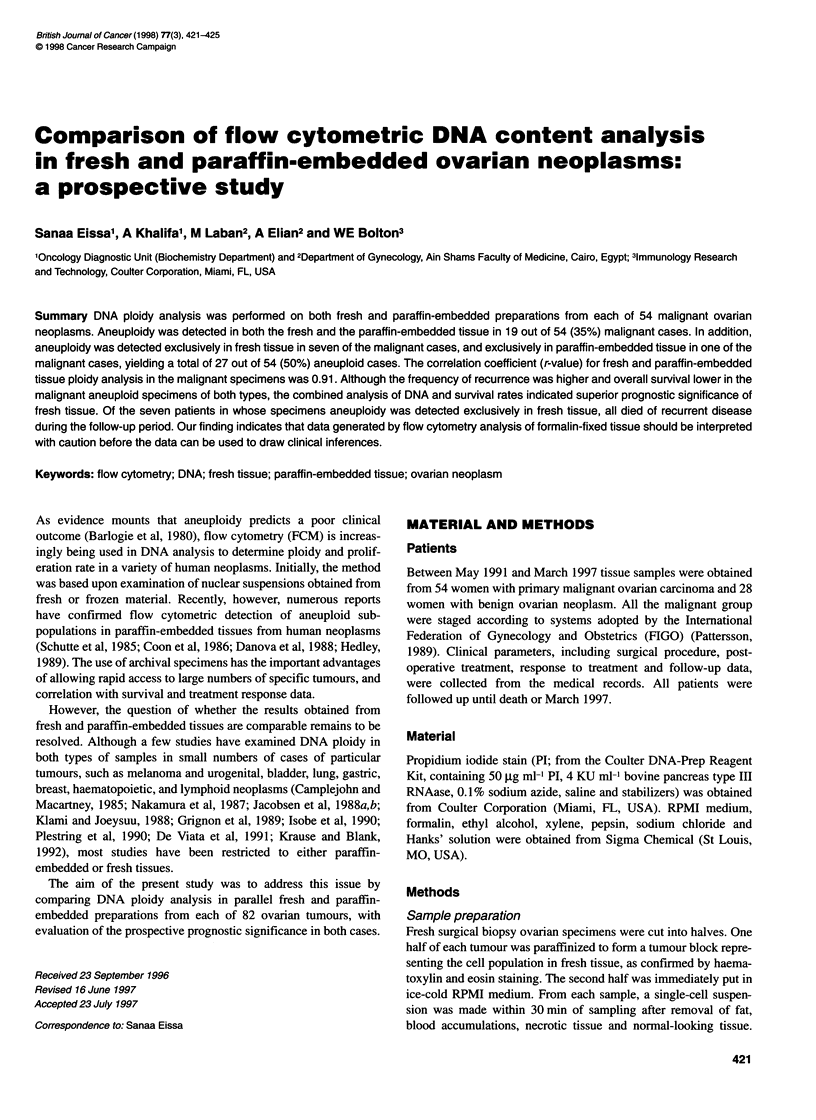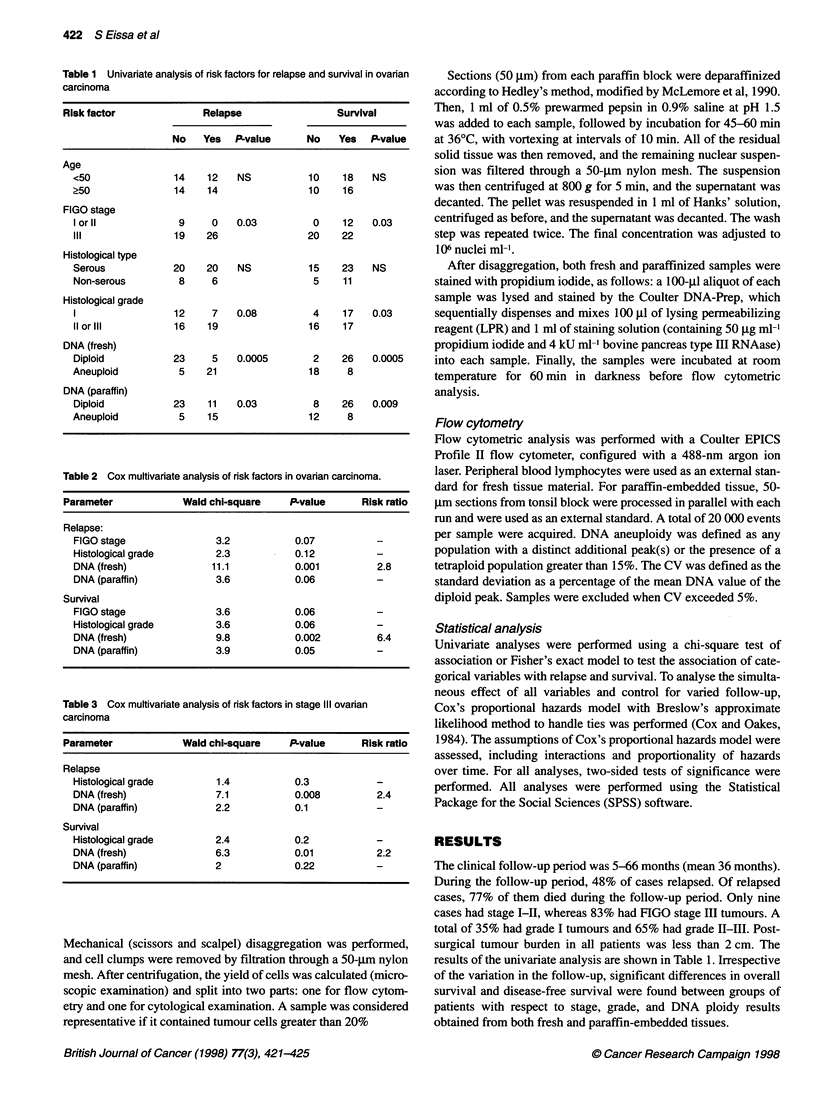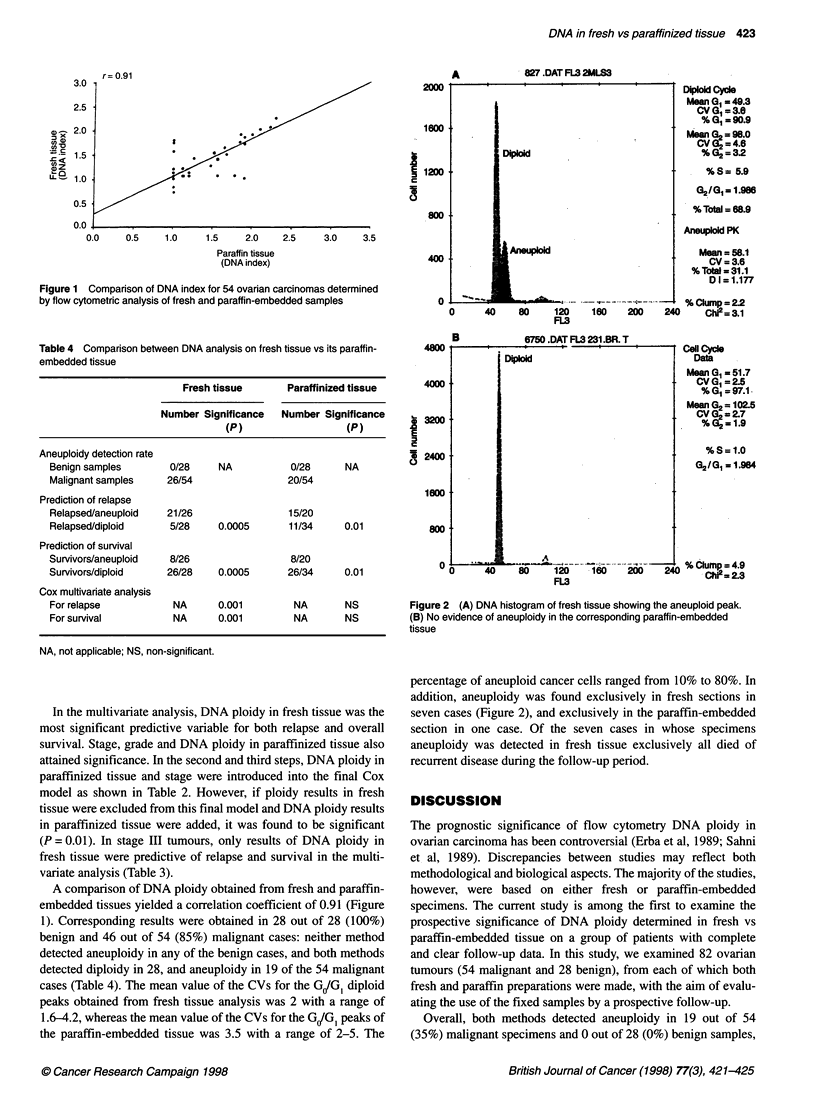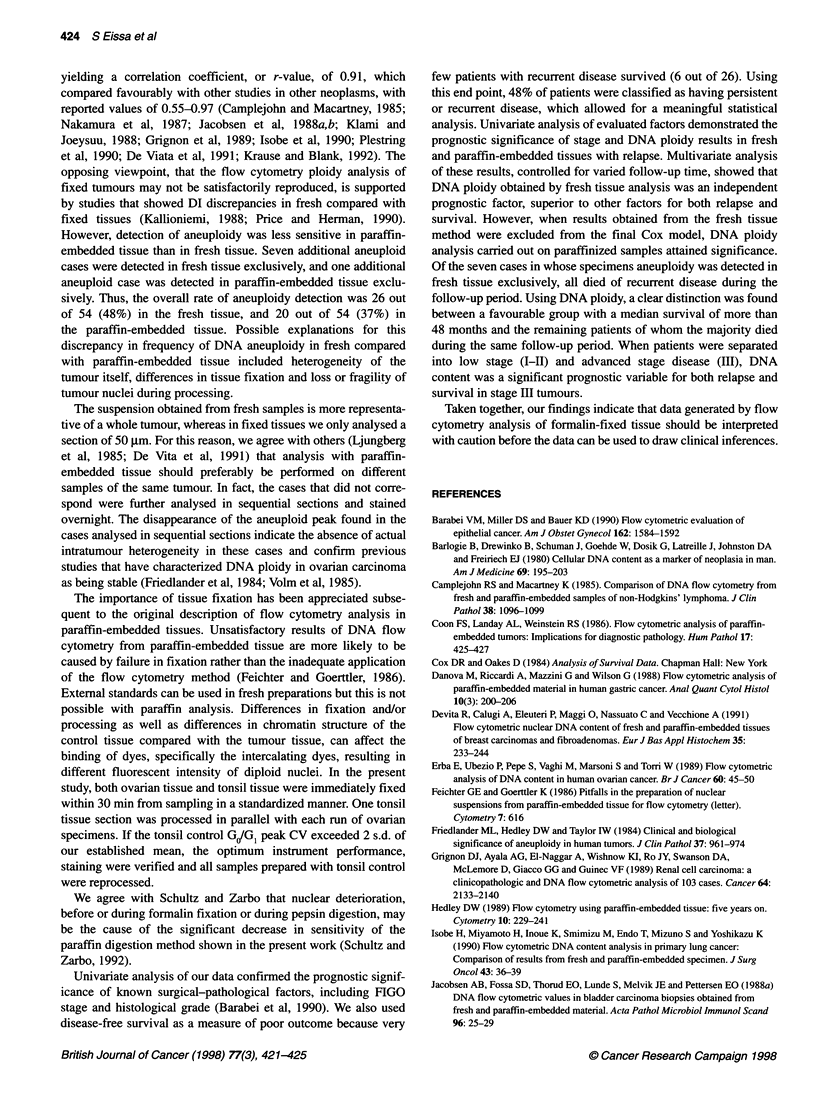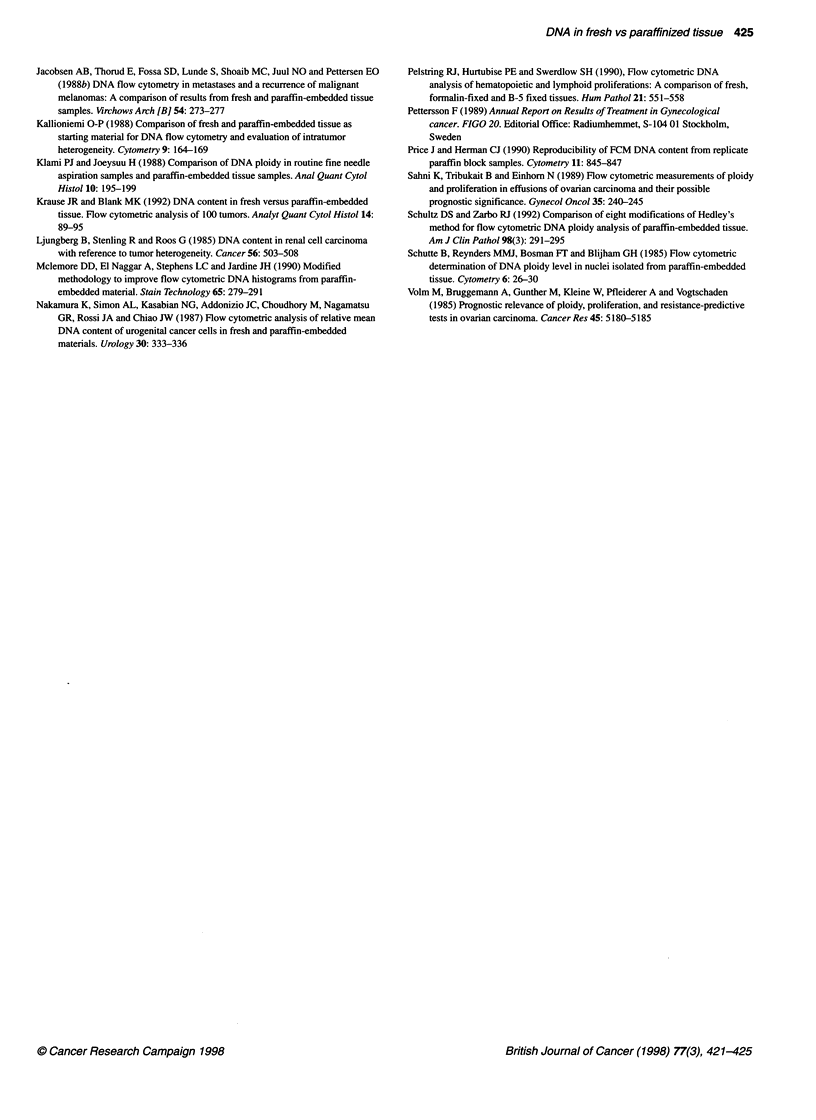# Comparison of flow cytometric DNA content analysis in fresh and paraffin-embedded ovarian neoplasms: a prospective study.

**DOI:** 10.1038/bjc.1998.67

**Published:** 1998

**Authors:** S. Eissa, A. Khalifa, M. Laban, A. Elian, W. E. Bolton

**Affiliations:** Oncology Diagnostic Unit (Biochemistry Department), Ain Shams Faculty of Medicine, Cairo, Egypt.

## Abstract

DNA ploidy analysis was performed on both fresh and paraffin-embedded preparations from each of 54 malignant ovarian neoplasms. Aneuploidy was detected in both the fresh and the paraffin-embedded tissue in 19 out of 54 (35%) malignant cases. In addition, aneuploidy was detected exclusively in fresh tissue in seven of the malignant cases, and exclusively in paraffin-embedded tissue in one of the malignant cases, yielding a total of 27 out of 54 (50%) aneuploid cases. The correlation coefficient (r-value) for fresh and paraffin-embedded tissue ploidy analysis in the malignant specimens was 0.91. Although the frequency of recurrence was higher and overall survival lower in the malignant aneuploid specimens of both types, the combined analysis of DNA and survival rates indicated superior prognostic significance of fresh tissue. Of the seven patients in whose specimens aneuploidy was detected exclusively in fresh tissue, all died of recurrent disease during the follow-up period. Our finding indicates that data generated by flow cytometry analysis of formalin-fixed tissue should be interpreted with caution before the data can be used to draw clinical inferences.


British Journal of Cancer (1998) 77(3), 421–425
© 1998 Cancer Research Campaign

### Comparison of flow cytometric DNA content analysis in fresh and paraffin-embedded ovarian neoplasms: a prospective study

**Sanaa Eissa[1], A Khalifa[1], M Laban[2], A Elian[2] and WE Bolton[3]**

[1]Oncology Diagnostic Unit (Biochemistry Department) and [2]Department of Gynecology, Ain Shams Faculty of Medicine, Cairo, Egypt; [3]Immunology Research and Technology, Coulter Corporation, Miami, FL, USA

**Summary** DNA ploidy analysis was performed on both fresh and paraffin-embedded preparations from each of 54 malignant ovarian neoplasms. Aneuploidy was detected in both the fresh and the paraffin-embedded tissue in 19 out of 54 (35%) malignant cases. In addition, aneuploidy was detected exclusively in fresh tissue in seven of the malignant cases, and exclusively in paraffin-embedded tissue in one of the malignant cases, yielding a total of 27 out of 54 (50%) aneuploid cases. The correlation coefficient (*r*-value) for fresh and paraffin-embedded tissue ploidy analysis in the malignant specimens was 0.91. Although the frequency of recurrence was higher and overall survival lower in the malignant aneuploid specimens of both types, the combined analysis of DNA and survival rates indicated superior prognostic significance of fresh tissue. Of the seven patients in whose specimens aneuploidy was detected exclusively in fresh tissue, all died of recurrent disease during the follow-up period. Our finding indicates that data generated by flow cytometry analysis of formalin-fixed tissue should be interpreted with caution before the data can be used to draw clinical inferences.

**Keywords:** flow cytometry; DNA; fresh tissue; paraffin-embedded tissue; ovarian neoplasm

As evidence mounts that aneuploidy predicts a poor clinical outcome (Barlogie et al, 1980), flow cytometry (FCM) is increasingly being used in DNA analysis to determine ploidy and proliferation rate in a variety of human neoplasms. Initially, the method was based upon examination of nuclear suspensions obtained from fresh or frozen material. Recently, however, numerous reports have confirmed flow cytometric detection of aneuploid subpopulations in paraffin-embedded tissues from human neoplasms (Schutte et al, 1985; Coon et al, 1986; Danova et al, 1988; Hedley, 1989). The use of archival specimens has the important advantages of allowing rapid access to large numbers of specific tumours, and correlation with survival and treatment response data.

However, the question of whether the results obtained from fresh and paraffin-embedded tissues are comparable remains to be resolved. Although a few studies have examined DNA ploidy in both types of samples in small numbers of cases of particular tumours, such as melanoma and urogenital, bladder, lung, gastric, breast, haematopoietic, and lymphoid neoplasms (Camplejohn and Macartney, 1985; Nakamura et al, 1987; Jacobsen et al, 1988*a,b*; Klami and Joeysuu, 1988; Grignon et al, 1989; Isobe et al, 1990; Plestring et al, 1990; De Viata et al, 1991; Krause and Blank, 1992), most studies have been restricted to either paraffin-embedded or fresh tissues.

The aim of the present study was to address this issue by comparing DNA ploidy analysis in parallel fresh and paraffin-embedded preparations from each of 82 ovarian tumours, with evaluation of the prospective prognostic significance in both cases.

*Received 23 September 1996*
*Revised 16 June 1997*
*Accepted 23 July 1997*

*Correspondence to:* Sanaa Eissa

### MATERIAL AND METHODS

### Patients

Between May 1991 and March 1997 tissue samples were obtained from 54 women with primary malignant ovarian carcinoma and 28 women with benign ovarian neoplasm. All the malignant group were staged according to systems adopted by the International Federation of Gynecology and Obstetrics (FIGO) (Pattersson, 1989). Clinical parameters, including surgical procedure, postoperative treatment, response to treatment and follow-up data, were collected from the medical records. All patients were followed up until death or March 1997.

### Material

Propidium iodide stain (PI; from the Coulter DNA-Prep Reagent Kit, containing 50 $\mu g\ ml^{-1}$ PI, 4 KU $ml^{-1}$ bovine pancreas type III RNAase, 0.1% sodium azide, saline and stabilizers) was obtained from Coulter Corporation (Miami, FL, USA). RPMI medium, formalin, ethyl alcohol, xylene, pepsin, sodium chloride and Hanks' solution were obtained from Sigma Chemical (St Louis, MO, USA).

### Methods

#### *Sample preparation*

Fresh surgical biopsy ovarian specimens were cut into halves. One half of each tumour was paraffinized to form a tumour block representing the cell population in fresh tissue, as confirmed by haematoxylin and eosin staining. The second half was immediately put in ice-cold RPMI medium. From each sample, a single-cell suspension was made within 30 min of sampling after removal of fat, blood accumulations, necrotic tissue and normal-looking tissue.

Table 1 Univariate analysis of risk factors for relapse and survival in ovarian carcinoma

| Risk factor | Relapse | | | Survival | | |
|---|---|---|---|---|---|---|
| | No | Yes | P-value | No | Yes | P-value |
| Age | | | | | | |
| <50 | 14 | 12 | NS | 10 | 18 | NS |
| ≥50 | 14 | 14 | | 10 | 16 | |
| FIGO stage | | | | | | |
| I or II | 9 | 0 | 0.03 | 0 | 12 | 0.03 |
| III | 19 | 26 | | 20 | 22 | |
| Histological type | | | | | | |
| Serous | 20 | 20 | NS | 15 | 23 | NS |
| Non-serous | 8 | 6 | | 5 | 11 | |
| Histological grade | | | | | | |
| I | 12 | 7 | 0.08 | 4 | 17 | 0.03 |
| II or III | 16 | 19 | | 16 | 17 | |
| DNA (fresh) | | | | | | |
| Diploid | 23 | 5 | 0.0005 | 2 | 26 | 0.0005 |
| Aneuploid | 5 | 21 | | 18 | 8 | |
| DNA (paraffin) | | | | | | |
| Diploid | 23 | 11 | 0.03 | 8 | 26 | 0.009 |
| Aneuploid | 5 | 15 | | 12 | 8 | |

Table 2 Cox multivariate analysis of risk factors in ovarian carcinoma.

| Parameter | Wald chi-square | P-value | Risk ratio |
|---|---|---|---|
| Relapse: | | | |
| FIGO stage | 3.2 | 0.07 | – |
| Histological grade | 2.3 | 0.12 | – |
| DNA (fresh) | 11.1 | 0.001 | 2.8 |
| DNA (paraffin) | 3.6 | 0.06 | – |
| Survival | | | |
| FIGO stage | 3.6 | 0.06 | – |
| Histological grade | 3.6 | 0.06 | – |
| DNA (fresh) | 9.8 | 0.002 | 6.4 |
| DNA (paraffin) | 3.9 | 0.05 | – |

Table 3 Cox multivariate analysis of risk factors in stage III ovarian carcinoma

| Parameter | Wald chi-square | P-value | Risk ratio |
|---|---|---|---|
| Relapse | | | |
| Histological grade | 1.4 | 0.3 | – |
| DNA (fresh) | 7.1 | 0.008 | 2.4 |
| DNA (paraffin) | 2.2 | 0.1 | – |
| Survival | | | |
| Histological grade | 2.4 | 0.2 | – |
| DNA (fresh) | 6.3 | 0.01 | 2.2 |
| DNA (paraffin) | 2 | 0.22 | – |

Mechanical (scissors and scalpel) disaggregation was performed, and cell clumps were removed by filtration through a 50-μm nylon mesh. After centrifugation, the yield of cells was calculated (microscopic examination) and split into two parts: one for flow cytometry and one for cytological examination. A sample was considered representative if it contained tumour cells greater than 20%

Sections (50 μm) from each paraffin block were deparaffinized according to Hedley's method, modified by McLemore et al, 1990. Then, 1 ml of 0.5% prewarmed pepsin in 0.9% saline at pH 1.5 was added to each sample, followed by incubation for 45–60 min at 36°C, with vortexing at intervals of 10 min. All of the residual solid tissue was then removed, and the remaining nuclear suspension was filtered through a 50-μm nylon mesh. The suspension was then centrifuged at 800 *g* for 5 min, and the supernatant was decanted. The pellet was resuspended in 1 ml of Hanks' solution, centrifuged as before, and the supernatant was decanted. The wash step was repeated twice. The final concentration was adjusted to $10^6$ nuclei $ml^{-1}$.

After disaggregation, both fresh and paraffinized samples were stained with propidium iodide, as follows: a 100-μl aliquot of each sample was lysed and stained by the Coulter DNA-Prep, which sequentially dispenses and mixes 100 μl of lysing permeabilizing reagent (LPR) and 1 ml of staining solution (containing 50 μg $ml^{-1}$ propidium iodide and 4 kU $ml^{-1}$ bovine pancreas type III RNAase) into each sample. Finally, the samples were incubated at room temperature for 60 min in darkness before flow cytometric analysis.

### *Flow cytometry*

Flow cytometric analysis was performed with a Coulter EPICS Profile II flow cytometer, configured with a 488-nm argon ion laser. Peripheral blood lymphocytes were used as an external standard for fresh tissue material. For paraffin-embedded tissue, 50-μm sections from tonsil block were processed in parallel with each run and were used as an external standard. A total of 20 000 events per sample were acquired. DNA aneuploidy was defined as any population with a distinct additional peak(s) or the presence of a tetraploid population greater than 15%. The CV was defined as the standard deviation as a percentage of the mean DNA value of the diploid peak. Samples were excluded when CV exceeded 5%.

### *Statistical analysis*

Univariate analyses were performed using a chi-square test of association or Fisher's exact model to test the association of categorical variables with relapse and survival. To analyse the simultaneous effect of all variables and control for varied follow-up, Cox's proportional hazards model with Breslow's approximate likelihood method to handle ties was performed (Cox and Oakes, 1984). The assumptions of Cox's proportional hazards model were assessed, including interactions and proportionality of hazards over time. For all analyses, two-sided tests of significance were performed. All analyses were performed using the Statistical Package for the Social Sciences (SPSS) software.

### RESULTS

The clinical follow-up period was 5–66 months (mean 36 months). During the follow-up period, 48% of cases relapsed. Of relapsed cases, 77% of them died during the follow-up period. Only nine cases had stage I–II, whereas 83% had FIGO stage III tumours. A total of 35% had grade I tumours and 65% had grade II–III. Post-surgical tumour burden in all patients was less than 2 cm. The results of the univariate analysis are shown in Table 1. Irrespective of the variation in the follow-up, significant differences in overall survival and disease-free survival were found between groups of patients with respect to stage, grade, and DNA ploidy results obtained from both fresh and paraffin-embedded tissues.

© Cancer Research Campaign 1998

**Figure 1** Comparison of DNA index for 54 ovarian carcinomas determined by flow cytometric analysis of fresh and paraffin-embedded samples

**Table 4** Comparison between DNA analysis on fresh tissue vs its paraffin-embedded tissue

| | **Fresh tissue** | | **Paraffinized tissue** | |
|---|---|---|---|---|
| | **Number** | **Significance (*P*)** | **Number** | **Significance (*P*)** |
| Aneuploidy detection rate | | | | |
| Benign samples | 0/28 | NA | 0/28 | NA |
| Malignant samples | 26/54 | | 20/54 | |
| Prediction of relapse | | | | |
| Relapsed/aneuploid | 21/26 | | 15/20 | |
| Relapsed/diploid | 5/28 | 0.0005 | 11/34 | 0.01 |
| Prediction of survival | | | | |
| Survivors/aneuploid | 8/26 | | 8/20 | |
| Survivors/diploid | 26/28 | 0.0005 | 26/34 | 0.01 |
| Cox multivariate analysis | | | | |
| For relapse | NA | 0.001 | NA | NS |
| For survival | NA | 0.001 | NA | NS |

NA, not applicable; NS, non-significant.

In the multivariate analysis, DNA ploidy in fresh tissue was the most significant predictive variable for both relapse and overall survival. Stage, grade and DNA ploidy in paraffinized tissue also attained significance. In the second and third steps, DNA ploidy in paraffinized tissue and stage were introduced into the final Cox model as shown in Table 2. However, if ploidy results in fresh tissue were excluded from this final model and DNA ploidy results in paraffinized tissue were added, it was found to be significant ($P = 0.01$). In stage III tumours, only results of DNA ploidy in fresh tissue were predictive of relapse and survival in the multivariate analysis (Table 3).

A comparison of DNA ploidy obtained from fresh and paraffin-embedded tissues yielded a correlation coefficient of 0.91 (Figure 1). Corresponding results were obtained in 28 out of 28 (100%) benign and 46 out of 54 (85%) malignant cases: neither method detected aneuploidy in any of the benign cases, and both methods detected diploidy in 28, and aneuploidy in 19 of the 54 malignant cases (Table 4). The mean value of the CVs for the $G_0/G_1$ diploid peaks obtained from fresh tissue analysis was 2 with a range of 1.6–4.2, whereas the mean value of the CVs for the $G_0/G_1$ peaks of the paraffin-embedded tissue was 3.5 with a range of 2–5. The

**Figure 2** (**A**) DNA histogram of fresh tissue showing the aneuploid peak. (**B**) No evidence of aneuploidy in the corresponding paraffin-embedded tissue percentage of aneuploid cancer cells ranged from 10% to 80%. In addition, aneuploidy was found exclusively in fresh sections in seven cases (Figure 2), and exclusively in the paraffin-embedded section in one case. Of the seven cases in whose specimens aneuploidy was detected in fresh tissue exclusively all died of recurrent disease during the follow-up period.

### DISCUSSION

The prognostic significance of flow cytometry DNA ploidy in ovarian carcinoma has been controversial (Erba et al, 1989; Sahni et al, 1989). Discrepancies between studies may reflect both methodological and biological aspects. The majority of the studies, however, were based on either fresh or paraffin-embedded specimens. The current study is among the first to examine the prospective significance of DNA ploidy determined in fresh vs paraffin-embedded tissue on a group of patients with complete and clear follow-up data. In this study, we examined 82 ovarian tumours (54 malignant and 28 benign), from each of which both fresh and paraffin preparations were made, with the aim of evaluating the use of the fixed samples by a prospective follow-up.

Overall, both methods detected aneuploidy in 19 out of 54 (35%) malignant specimens and 0 out of 28 (0%) benign samples,

*© Cancer Research Campaign 1998* 

yielding a correlation coefficient, or *r*-value, of 0.91, which compared favourably with other studies in other neoplasms, with reported values of 0.55–0.97 (Camplejohn and Macartney, 1985; Nakamura et al, 1987; Jacobsen et al, 1988*a,b*; Klami and Joeysuu, 1988; Grignon et al, 1989; Isobe et al, 1990; Plestring et al, 1990; De Viata et al, 1991; Krause and Blank, 1992). The opposing viewpoint, that the flow cytometry ploidy analysis of fixed tumours may not be satisfactorily reproduced, is supported by studies that showed DI discrepancies in fresh compared with fixed tissues (Kallioniemi, 1988; Price and Herman, 1990). However, detection of aneuploidy was less sensitive in paraffin-embedded tissue than in fresh tissue. Seven additional aneuploid cases were detected in fresh tissue exclusively, and one additional aneuploid case was detected in paraffin-embedded tissue exclusively. Thus, the overall rate of aneuploidy detection was 26 out of 54 (48%) in the fresh tissue, and 20 out of 54 (37%) in the paraffin-embedded tissue. Possible explanations for this discrepancy in frequency of DNA aneuploidy in fresh compared with paraffin-embedded tissue included heterogeneity of the tumour itself, differences in tissue fixation and loss or fragility of tumour nuclei during processing.

The suspension obtained from fresh samples is more representative of a whole tumour, whereas in fixed tissues we only analysed a section of 50 μm. For this reason, we agree with others (Ljungberg et al, 1985; De Vita et al, 1991) that analysis with paraffin-embedded tissue should preferably be performed on different samples of the same tumour. In fact, the cases that did not correspond were further analysed in sequential sections and stained overnight. The disappearance of the aneuploid peak found in the cases analysed in sequential sections indicate the absence of actual intratumour heterogeneity in these cases and confirm previous studies that have characterized DNA ploidy in ovarian carcinoma as being stable (Friedlander et al, 1984; Volm et al, 1985).

The importance of tissue fixation has been appreciated subsequent to the original description of flow cytometry analysis in paraffin-embedded tissues. Unsatisfactory results of DNA flow cytometry from paraffin-embedded tissue are more likely to be caused by failure in fixation rather than the inadequate application of the flow cytometry method (Feichter and Goerttler, 1986). External standards can be used in fresh preparations but this is not possible with paraffin analysis. Differences in fixation and/or processing as well as differences in chromatin structure of the control tissue compared with the tumour tissue, can affect the binding of dyes, specifically the intercalating dyes, resulting in different fluorescent intensity of diploid nuclei. In the present study, both ovarian tissue and tonsil tissue were immediately fixed within 30 min from sampling in a standardized manner. One tonsil tissue section was processed in parallel with each run of ovarian specimens. If the tonsil control $G_0/G_1$ peak CV exceeded 2 s.d. of our established mean, the optimum instrument performance, staining were verified and all samples prepared with tonsil control were reprocessed.

We agree with Schultz and Zarbo that nuclear deterioration, before or during formalin fixation or during pepsin digestion, may be the cause of the significant decrease in sensitivity of the paraffin digestion method shown in the present work (Schultz and Zarbo, 1992).

Univariate analysis of our data confirmed the prognostic significance of known surgical–pathological factors, including FIGO stage and histological grade (Barabei et al, 1990). We also used disease-free survival as a measure of poor outcome because very few patients with recurrent disease survived (6 out of 26). Using this end point, 48% of patients were classified as having persistent or recurrent disease, which allowed for a meaningful statistical analysis. Univariate analysis of evaluated factors demonstrated the prognostic significance of stage and DNA ploidy results in fresh and paraffin-embedded tissues with relapse. Multivariate analysis of these results, controlled for varied follow-up time, showed that DNA ploidy obtained by fresh tissue analysis was an independent prognostic factor, superior to other factors for both relapse and survival. However, when results obtained from the fresh tissue method were excluded from the final Cox model, DNA ploidy analysis carried out on paraffinized samples attained significance. Of the seven cases in whose specimens aneuploidy was detected in fresh tissue exclusively, all died of recurrent disease during the follow-up period. Using DNA ploidy, a clear distinction was found between a favourable group with a median survival of more than 48 months and the remaining patients of whom the majority died during the same follow-up period. When patients were separated into low stage (I–II) and advanced stage disease (III), DNA content was a significant prognostic variable for both relapse and survival in stage III tumours.

Taken together, our findings indicate that data generated by flow cytometry analysis of formalin-fixed tissue should be interpreted with caution before the data can be used to draw clinical inferences.

### REFERENCES

Barabei VM, Miller DS and Bauer KD (1990) Flow cytometric evaluation of epithelial cancer. *Am J Obstet Gynecol* **162**: 1584–1592

Barlogie B, Drewinko B, Schuman J, Goehde W, Dosik G, Latreille J, Johnston DA and Freiriech EJ (1980) Cellular DNA content as a marker of neoplasia in man. *Am J Medicine* **69**: 195–203

Camplejohn RS and Macartney K (1985). Comparison of DNA flow cytometry from fresh and paraffin-embedded samples of non-Hodgkins' lymphoma. *J Clin Pathol* **38**: 1096–1099

Coon FS, Landay AL, Weinstein RS (1986). Flow cytometric analysis of paraffin-embedded tumors: Implications for diagnostic pathology. *Hum Pathol* **17**: 425–427

Cox DR and Oakes D (1984) *Analysis of Survival Data*. Chapman Hall: New York

Danova M, Riccardi A, Mazzini G and Wilson G (1988) Flow cytometric analysis of paraffin-embedded material in human gastric cancer. *Anal Quant Cytol Histol* **10**(3): 200–206

Devita R, Calugi A, Eleuteri P, Maggi O, Nassuato C and Vecchione A (1991) Flow cytometric nuclear DNA content of fresh and paraffin-embedded tissues of breast carcinomas and fibroadenomas. *Eur J Bas Appl Histochem* **35**: 233–244

Erba E, Ubezio P, Pepe S, Vaghi M, Marsoni S and Torri W (1989) Flow cytometric analysis of DNA content in human ovarian cancer. *Br J Cancer* **60**: 45–50

Feichter GE and Goerttler K (1986) Pitfalls in the preparation of nuclear suspensions from paraffin-embedded tissue for flow cytometry (letter). *Cytometry* **7**: 616

Friedlander ML, Hedley DW and Taylor IW (1984) Clinical and biological significance of aneuploidy in human tumors. *J Clin Pathol* **37**: 961–974

Grignon DJ, Ayala AG, El-Naggar A, Wishnow KI, Ro JY, Swanson DA, McLemore D, Giacco GG and Guinec VF (1989) Renal cell carcinoma: a clinicopathologic and DNA flow cytometric analysis of 103 cases. *Cancer* **64**: 2133–2140

Hedley DW (1989) Flow cytometry using paraffin-embedded tissue: five years on. *Cytometry* **10**: 229–241

Isobe H, Miyamoto H, Inoue K, Smimizu M, Endo T, Mizuno S and Yoshikazu K (1990) Flow cytometric DNA content analysis in primary lung cancer: Comparison of results from fresh and paraffin-embedded specimen. *J Surg Oncol* **43**: 36–39

Jacobsen AB, Fossa SD, Thorud EO, Lunde S, Melvik JE and Pettersen EO (1988*a*) DNA flow cytometric values in bladder carcinoma biopsies obtained from fresh and paraffin-embedded material. *Acta Pathol Microbiol Immunol Scand* **96**: 25–29

© Cancer Research Campaign 1998

Jacobsen AB, Thorud E, Fossa SD, Lunde S, Shoaib MC, Juul NO and Pettersen EO (1988*b*) DNA flow cytometry in metastases and a recurrence of malignant melanomas: A comparison of results from fresh and paraffin-embedded tissue samples. *Virchows Arch [B]* **54**: 273–277

Kallioniemi O-P (1988) Comparison of fresh and paraffin-embedded tissue as starting material for DNA flow cytometry and evaluation of intratumor heterogeneity. *Cytometry* **9**: 164–169

Klami PJ and Joeysuu H (1988) Comparison of DNA ploidy in routine fine needle aspiration samples and paraffin-embedded tissue samples. *Anal Quant Cytol Histol* **10**: 195–199

Krause JR and Blank MK (1992) DNA content in fresh versus paraffin-embedded tissue. Flow cytometric analysis of 100 tumors. *Analyt Quant Cytol Histol* **14**: 89–95

Ljungberg B, Stenling R and Roos G (1985) DNA content in renal cell carcinoma with reference to tumor heterogeneity. *Cancer* **56**: 503–508

Mclemore DD, El Naggar A, Stephens LC and Jardine JH (1990) Modified methodology to improve flow cytometric DNA histograms from paraffin-embedded material. *Stain Technology* **65**: 279–291

Nakamura K, Simon AL, Kasabian NG, Addonizio JC, Choudhory M, Nagamatsu GR, Rossi JA and Chiao JW (1987) Flow cytometric analysis of relative mean DNA content of urogenital cancer cells in fresh and paraffin-embedded materials. *Urology* **30**: 333–336

Pelstring RJ, Hurtubise PE and Swerdlow SH (1990), Flow cytometric DNA analysis of hematopoietic and lymphoid proliferations: A comparison of fresh, formalin-fixed and B-5 fixed tissues. *Hum Pathol* **21**: 551–558

Pettersson F (1989) *Annual Report on Results of Treatment in Gynecological cancer. FIGO 20*. Editorial Office: Radiumhemmet, S-104 01 Stockholm, Sweden

Price J and Herman CJ (1990) Reproducibility of FCM DNA content from replicate paraffin block samples. *Cytometry* **11**: 845–847

Sahni K, Tribukait B and Einhorn N (1989) Flow cytometric measurements of ploidy and proliferation in effusions of ovarian carcinoma and their possible prognostic significance. *Gynecol Oncol* **35**: 240–245

Schultz DS and Zarbo RJ (1992) Comparison of eight modifications of Hedley's method for flow cytometric DNA ploidy analysis of paraffin-embedded tissue. *Am J Clin Pathol* **98**(3): 291–295

Schutte B, Reynders MMJ, Bosman FT and Blijham GH (1985) Flow cytometric determination of DNA ploidy level in nuclei isolated from paraffin-embedded tissue. *Cytometry* **6**: 26–30

Volm M, Bruggemann A, Gunther M, Kleine W, Pfleiderer A and Vogtschaden (1985) Prognostic relevance of ploidy, proliferation, and resistance-predictive tests in ovarian carcinoma. *Cancer Res* **45**: 5180–5185

© Cancer Research Campaign 1998